# Bispecific antibody CAP256.J3LS targets V2-apex and CD4-binding sites with high breadth and potency

**DOI:** 10.1080/19420862.2023.2165390

**Published:** 2023-02-02

**Authors:** Baoshan Zhang, Jason Gorman, Young D. Kwon, Amarendra Pegu, Cara W. Chao, Tracy Liu, Mangaiarkarasi Asokan, Michael F. Bender, Tatsiana Bylund, Leland Damron, Deepika Gollapudi, Paula Lei, Yile Li, Cuiping Liu, Mark K. Louder, Krisha McKee, Adam S. Olia, Reda Rawi, Arne Schön, Shuishu Wang, Eun Sung Yang, Yongping Yang, Kevin Carlton, Nicole A. Doria-Rose, Lawrence Shapiro, Michael S. Seaman, John R. Mascola, Peter D. Kwong

**Affiliations:** a Vaccine Research Center, NIAID, National Institutes of Health, Bethesda, MD, USA; b Department of Biology, Johns Hopkins University, Baltimore, MD, USA; c Department of Biochemistry, Columbia University, New York, NY, USA; d Beth Israel Deaconess Medical Center, Harvard Medical School, Boston, MA, USA

**Keywords:** Antibody half-life, antibody improvement, bispecific antibody, CAP256-VRC26.25, CD4-binding site, cryo-electron microscopy, HIV-1, J3 nanobody, V2 apex

## Abstract

Antibody CAP256-VRC26.25 targets the second hypervariable region (V2) at the apex of the HIV envelope (Env) trimer with extraordinary neutralization potency, although less than optimal breadth. To improve breadth, we linked the light chain of CAP256V2LS, an optimized version of CAP256-VRC26.25 currently under clinical evaluation, to the llama nanobody J3, which has broad CD4-binding site-directed neutralization. The J3-linked bispecific antibody exhibited improved breadth and potency over both J3 and CAP256V2LS, indicative of synergistic neutralization. The cryo-EM structure of the bispecific antibody in complex with a prefusion-closed Env trimer revealed simultaneous binding of J3 and CAP256V2LS. We further optimized the pharmacokinetics of the bispecific antibody by reducing the net positive charge of J3. The optimized bispecific antibody, which we named CAP256.J3LS, had a half-life similar to CAP256V2LS in human FcRn knock-in mice and exhibited suitable auto-reactivity, manufacturability, and biophysical risk. CAP256.J3LS neutralized over 97% of a multiclade 208-strain panel (geometric mean concentration for 80% inhibition (IC_80_) 0.079 μg/ml) and 100% of a 100-virus clade C panel (geometric mean IC_80_ of 0.05 μg/ml), suggesting its anti-HIV utility especially in regions where clade C dominates.

## Introduction

Antibody-mediated prevention (AMP) of HIV-1 infection has been a long-sought goal. AMP clinical studies of VRC01 demonstrate the ability of passively delivered antibodies to prevent HIV-1 infection,^1^ but prevention by VRC01 requires a neutralization potency of better than 1 μg/ml 80% of maximal inhibition concentration (IC_80_). This result has set off a search for other broadly neutralizing antibodies, capable of neutralizing at this potency.

Antibodies against the second hypervariable region (V2), at the trimer apex, are among the most potent broadly neutralizing antibodies identified to date;^2^ on a 208-strain panel, V2-apex-directed antibodies such as PG9 and PG16^3^ neutralize at a mean IC_80_ of 0.34 and 0.113 μg/ml, respectively, PGT145^4^ and its somatic variant PGDM1400^5^ neutralizes at 0.343 and 0.049 μg/ml, respectively, and CAP256-VRC26.25^6^ neutralizes at 0.035 μg/ml. However, the neutralization breadths of these antibodies have been less than ideal, ranging from 50% to 80% on our 208-strain panel. Multispecific antibodies could improve both breadth and potency, if the right combination could be identified (along with the right linker) to enable simultaneous engagement of multiple sites of vulnerability, including the potent V2 apex site and other sites more conserved among multiple clade viral strains.

Structures of PG9, PGT145, and CAP256-VRC26.25 in complex with HIV-1 envelope (Env) trimer^7,8^ or V1V2 scaffolds^9^ reveal recognition to occur via an extended 3^rd^ heavy chain complementarity-determining region (CDR H3), leaving the light chain relatively unencumbered, a potentially ready site for conjugation. Indeed, among the over 2000 non-redundant structures of antibodies bound to antigen, V2-apex-directed antibodies have the longest distance between antibody-framework and antigen, with CAP256-VRC26.25 being the furthest from the antigen.^10^ One strategy to design bispecific antibodies is to link the light chain of these V2-apex-directed antibodies to a broadly neutralizing antibody targeting conserved sites other than the V2 site of vulnerability. The membrane-proximal external region (MPER) is well conserved and MPER-directed antibodies, such as 10E8, are very broad and potent.^11^ CD4-binding site-directed antibodies are suitable for fusion to V2-apex-directed antibodies as bispecific antibodies because the CD4-binding site is close to V2-apex and is more conserved. Several broadly neutralizing antibodies and nanobodies targeting CD4-binding sites, such as VRC01^12,13^ and J3,^14,15^ are available.

Here, we generated bispecific antibodies by genetically fusing single-chain variable regions (scFvs) of human antibodies or single variable domains of heavy-chain-only antibodies (nanobodies) to the light chain N terminus of CAP256V2LS, an optimized version of CAP256-VRC26.25. We assessed binding and neutralization for these bispecific antibodies and structurally characterized the broadest and most potent. We improved pharmacokinetics of these bispecific antibodies by reducing their surface electropositivity and assessed their physical properties, neutralization, manufacturability, and biophysical risk. Overall, we identify CAP256.J3LS, a bispecific antibody with a suitable half-life, capable of neutralizing 97% of a 208-virus multiclade panel and 100% of a clade C panel at an IC_80_ of less than 50 μg/ml, and 77% of the 208-strain panel and 82% of the clade C panel at IC_80_ of less than 1 μg/ml.

## Results

### Design of bispecific antibodies

**Figure 1.**
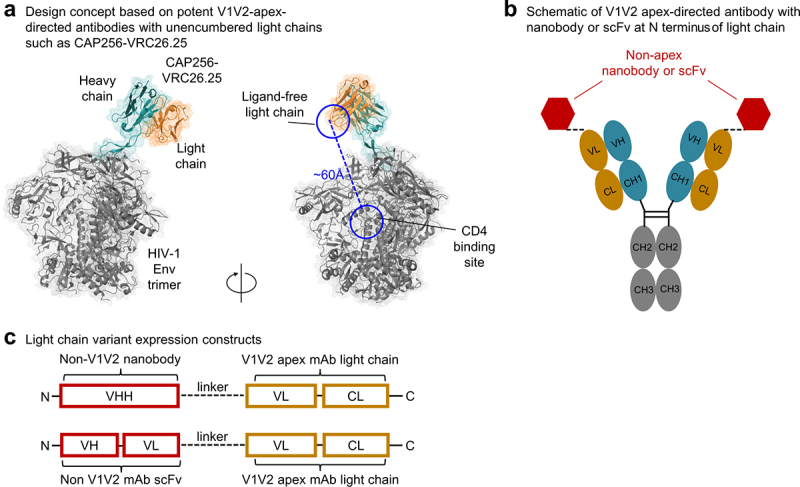
Design of HIV-1 bispecific antibodies attaching nanobodies or scFvs to the light chain of the super potent V2-apex-directed antibody CAP256-VRC26.25. (a) Structure of the antigen-binding fragment (Fab) of antibody CAP256-VRC26.25 in complex with a prefusion-closed HIV-1 Env trimer showing an unencumbered light chain allowing its linkage to other HIV-1 trimer-binding components. The resultant bispecific antibody enables synergistic binding and enhanced neutralization. (b) Schematic of V1V2 antibody with additional binding component genetically fused to the light chain N terminus. (c) Light chain variant expression constructs. Sequence information of these variants are listed in supplementary figures. Upper construct shows light chain variants linked with nanobody. Bottom construct shows light chain variants linked to single-chain Fv.

The published structure of the super-potent antibody CAP256-VRC26.25^6^ in complex with a prefusion-closed envelope trimer^7^ reveals that the light chain of this antibody does not interact directly with Env, leaving its N terminus free for conjugation (Figure 1a,b). We designed a number of bispecific antibodies by genetically fusing the light chain N terminus of CAP256V2LS, which is an optimized version of CAP256-VRC26.25 with K100mA and LS mutations recently assessed in clinical trials,^16–18^ with nanobody J3 or scFv antibodies 10E8 and VRC01 that target non-V1V2 sites (Figure 1c and Supplementary Table S1).

**Figure 2.**
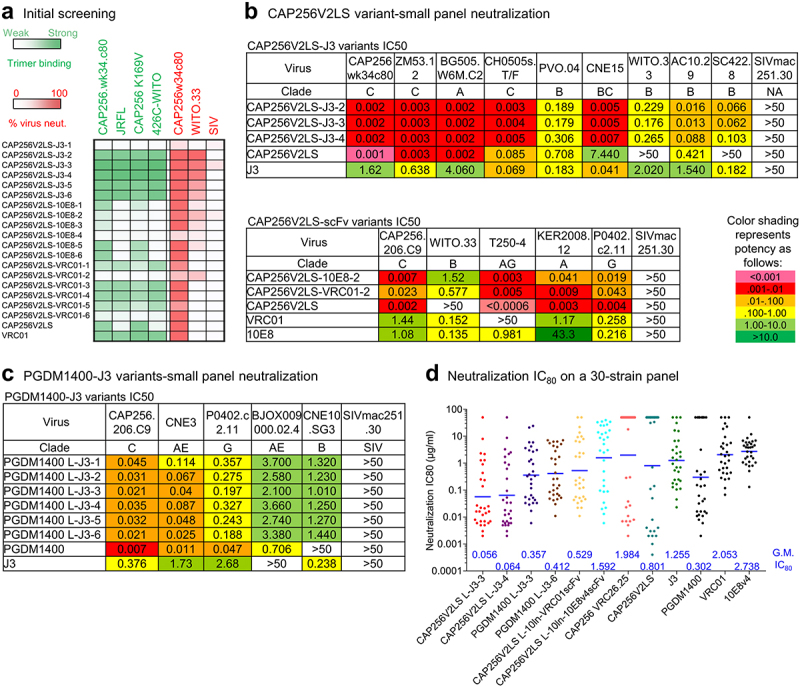
Evaluation of antibody variants and selection of variants with improved neutralizing breadth. (a) Screening of designed antibody variants for binding to trimers from CAP256V2LS-resistant virus strains and neutralization on a CAP256V2LS-resistant virus. Each row represent the results of binding in green color or neutralization in red color. Darker colors indicate better binding or neutralization. (b) Neutralization IC_50_ of CAP256V2LS nanobody variants and scFv variants on small virus panels. (c) Neutralization IC_50_ of PGDM1400 antibody variants on a 5-virus panel. (d) Neutralization IC_80_ of selected CAP256V2LS and PGDM1400 antibody variants on a 30-virus panel. Geometric mean IC_80_ values (µg/ml) are indicated at the bottom of each antibody column.

We synthesized, expressed, and tested these light-chain variants of CAP256V2LS for binding to soluble Env trimers stabilized in the prefusion-closed confirmation by automated design and consensus repair.^19^ Tested strains included CAP256 (from clade C), JRFL (B), 426C-WITO (B), as well as a K169V variant of CAP256 with reduced binding of CAP256-VRC26.25; we also tested the ability of the light-chain variants to neutralize CAP256 and WITO.33, with simian immunodeficiency virus (SIV) as a negative control (Figure 2a, Supplementary Figure S1). We found several light-chain variants with J3 fusion to bind tightly Env trimers of tested strains and to neutralize both CAP256 and WITO.33, but not SIV. The best J3 variants (CAP256V2LS-J3-2, −3, and −4) used linkers of 2x GGSGG, 3x GGSGG, and 1x DKTHT, respectively (Supplementary Fig. 1 and Supplementary Table 1), and we further tested these on a nine-strain panel, including WITO.33 and SC422.8, which were resistant to CAP256-VRC26.25, but moderately sensitive to J3. Notably, the neutralization potency of CAP256V2LS-J3-2 and CAP256V2LS-J3-3, whose linkers were 10- and 15-residues, respectively, was ~10-fold better against WITO.33 and 3-fold against SC422.8 than that of J3 (Figure 2b). Neutralization potencies against strains modestly neutralized by CAP256V2LS were also substantially improved for these two bispecific antibodies, relative to that of both CAP256V2LS and J3. Improved neutralization appeared to be specific to the bispecific antibodies with J3 fusion and was not observed with bispecific antibodies constructed from scFvs of VRC01 or 10E8. We attribute the lower efficacy of scFv fusion to the larger size of scFv versus nanobody; the substantially smaller size of the nanobody likely enables it to reach the CD4 binding site, while simultaneously allows recognition at the V2 apex site (Figure 2b).

To test if J3 fusion to the N terminus of light chain could improve other V2 apex antibodies, we tried the same strategy with PGDM1400 using six linkers ranging in length from 5 to 15 residues (Figure 2c); all six of the PGDM1400-J3 variants showed weaker neutralization than either PGDM1400 or J3, suggesting that unlike the CAP256V2LS-J3 fusions, the PGDM1400-J3 variants could not simultaneously recognize both epitopes, likely because the light chain N terminus, the site of attachment for the nanobody, is substantially closer to V2 apex in the case of PGDM1400 versus that of CAP256V2LS.

Neutralization assessment on a 30-strain panel indicated the CAP256V2LS-J3-3 variant to be the best, slightly better than CAP256V2LS-J3-4 variant, and substantially better than other bispecific antibodies with PGDM1400 or CAP256V2LS with scFvs (Figure 2d; Supplementary Fig. 2).

### Structure of CAP256V2LS-J3-3 in complex with BG505 DS-SOSIP.664

**Figure 3.**
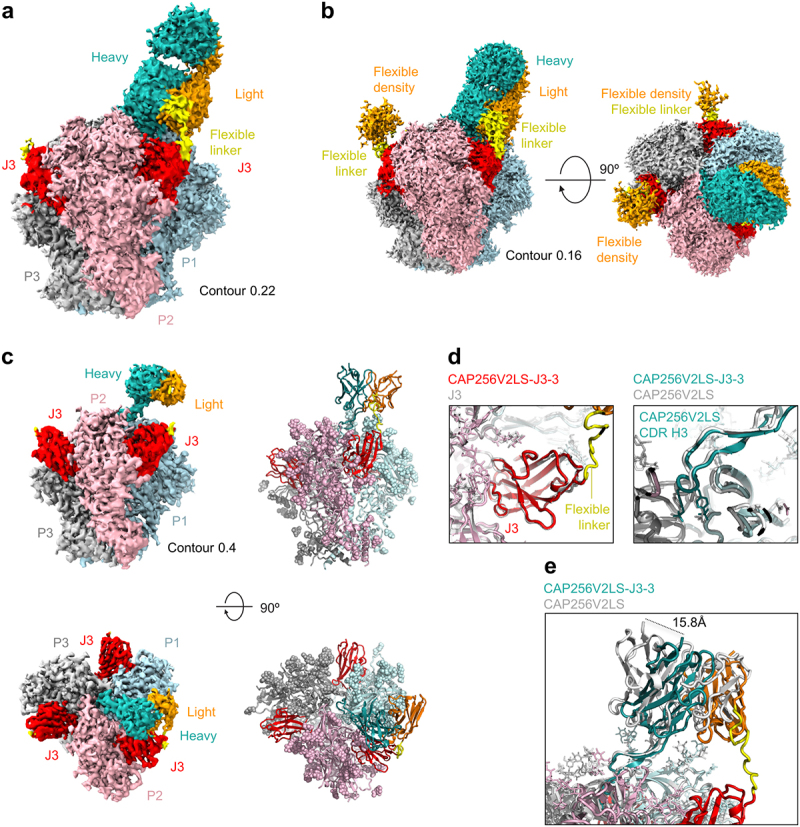
Structure of CAP256V2LS-J3-3 Fab in complex with BG505 DS-SOSIP.664 confirms avidity and stoichiometry. (a) Cryo-EM density is shown highlighting the linker between CAP256V2LS and J3 at 3.2 Å resolution and contour of 0.22 where density for the flexible linker was visible. (b) Cryo-EM density is shown with lower contour to illustrate the signal observed corresponding to unbound CAP256V2LS in Orange (c) (left) Cryo-EM density is shown with higher contour revealing greater detail of higher resolution signal. (right) Corresponding atomic models are shown in cartoon format with glycans shown as spheres. (d) Details of binding for CAP256V2LS and J3 from the bivalent complex structure are overlayed with the structures obtained from individual components. (e) The CDR H3 of CAP256V2LS aligns closely while the main body of the Fab shifts as much as ~16 Å.

To elucidate the mechanism of the synergistic binding and neutralization of the CAP256V2LS-J3 bispecific antibody, we determined the cryo-EM structure of CAP256V2LS-J3-3 antigen-binding fragment (Fab) in complex with BG505 DS-SOSIP.644 trimer^20,21^ at 3.2 Å resolution (Figure 3a; Supplementary Figure S3; Supplementary Table S2). We mixed CAP256V2LS-J3-3 with BG505 DS-SOSIP.664 trimer at a 3.5:1 molar ratio for cryo-EM grid preparation. As expected, a single CAP256V2LS Fab was observed to bind at the apex hole formed by the three protomers of the trimer, as observed previously for the CAP256-VRC26.25 Fab in a complex with Env trimer.^7^ Clear density was present for the linker connecting CAP256V2LS light chain N terminus to the C terminus of J3 binding at the most proximal CD4-binding site. At each of the other two CD4-binding sites of the trimer, J3 was observed to bind, with weak density extending from its C terminus, but the linked CAP256V2LS was not visible (Figure 3b). The linker density was mostly missing at a higher contour level (Figure 3c), indicating that the linker for these two molecules was flexible. At this contour level, the density for the constant domains of CAP256V2LS bound at the trimer apex was also not visible, and only its variable domains could be seen (Figure 3c). Binding of J3 generally recapitulated the binding mode observed for J3 alone, as did the CDR H3 of CAP256-VRC26.25 (Figure 3d); however, the variable domain swiveled, so that the C terminus of the heavy chain was displaced almost 16 Å (Figure 3e).

Overall, the structure confirmed the ability of the CAP256V2LS-J3-3 to bind simultaneously to both V2 apex and CD4-binding site, with two other copies of the bispecific antibody binding through only their J3 moieties to the other two CD4-binding sites of the trimer.

### Improved pharmacokinetics of CAP256.J3LS achieved by altering surface charge of J3

**Figure 4.**
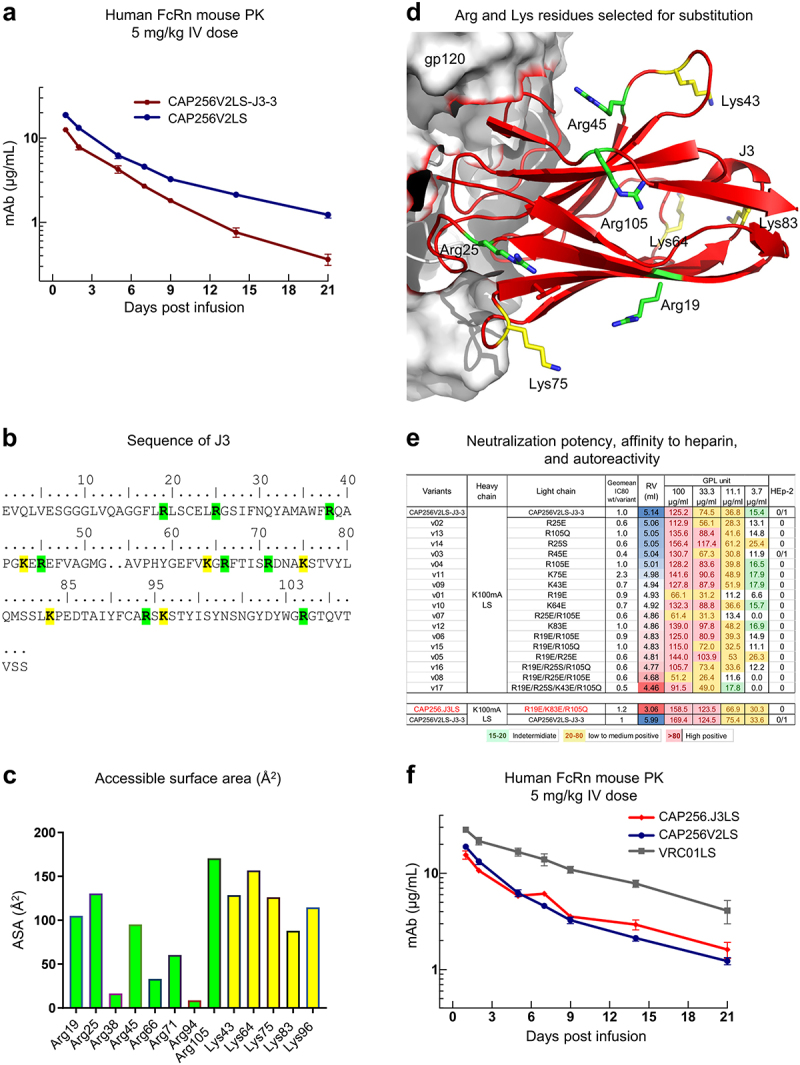
Improved pharmacokinetics of CAP256.J3LS achieved by altering surface charge of J3. (a) *In vivo* half-life of CAP256- variants assessed in a human FcRn knock-in mouse model. (b) Sequence of J3 with Arg and Lys residues highlighted, and J3 paratope residues are underlined. (c) Accessible surface area of Arg and Lys residues. Residues above the dotted line were altered mutationally to reduce electropositivity. (d) Arg and Lys residues that were selected for mutations were shown in the structure of J3 in complex with gp120 (PDB ID: 7RI1). (e) Neutralization IC80 fold change, heparin chromatography retention volume, and autoreactivity of CAP256.J3LS variants. (f) *In vivo* half-life of CAP256.J3LS variants.

We compared the pharmacokinetics of CAP256V2LS-J3-3 with its parent antibody CAP256V2LS.^16,18^ We observed the bispecific antibody to have reduced half-life relative to CAP256V2LS in neonatal Fc receptor (FcRn)-knock-in mice (Figure 4a; Supplementary Table S3). As the degree of electropositivity of an antibody variable domain has been observed to correlate inversely with half-life,^22–24^ we sought to decrease the electropositivity of J3 by mutating surface-exposed Lys and Arg to Glu or other less positively charged amino acids, as we have recently found that this procedure extends the half-life of CD4-binding site antibodies (YDK, personal communication). We analyzed the surface accessibility of Lys and Arg residues on J3 and mutated the surface-exposed residues one at a time or in combinations (Figure 4b-e). Several variants showed similar IC_80_ to that of CAP256V2LS, with lower retention volume as measured by heparin affinity chromatography (Figure 4e; Supplementary Figure S4), and improved pharmacokinetics in FcRn mouse, with half-life predicted to be similar or perhaps even longer than CAP256V2LS. The best variant, named CAP256.J3LS, had mutations in J3 of R19E, K83E, and R105Q, and low autoreactivity, good neutralization IC_80_s, and decent pharmacokinetics in FcRn-KI mice (Figure 4f, Supplementary Figure S5).

### CAP256.J3LS properties

The CAP256.J3LS bispecific antibody expressed well in mammalian cells (Supplementary Figure S6) and could be purified following the same steps as its parent antibody CAP256V2LS, as the additional J3 component was only attached to the light chain. Isothermal titration calorimetry (ITC) with BG505 DS-SOSIP.664 showed improved affinity for CAP256.J3LS relative to CAP256V2LS and J3 (Supplementary Figure S7).

**Figure 5.**
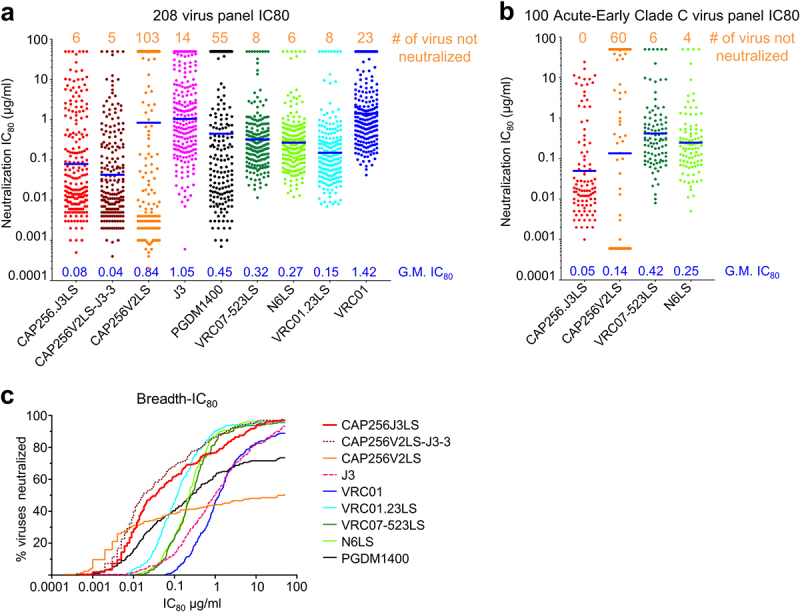
Neutralization breath and potency of CAP256.J3LS antibody. (a) Neutralization IC_80_ of CAP256.J3LS on a 208 global virus panel in comparison with parental antibodies and select HIV-1 antibodies in clinical development. (b) Neutralization IC_80_ of CAP256.J3LS on a 100 Acute-Early Clade C virus panel. (c) Breadth-IC_80_ curves of CAP256.J3LS in comparison with parental antibodies and select HIV-1 antibodies in clinical development.

We assessed the neutralization on a 208-strain panel (Figure 5a) and a 100-strain clade-C-specific panel (Figure 5b). On the 208-strain panel, CAP256.J3LS showed neutralization potencies that were substantially superior to other broad neutralizers, including PGDM1400 and N6LS, neutralizing 97% with a geometric mean IC_80_ of 0.08 μg/ml (Figure 5a; Supplementary Table S4). Interestingly, the analysis of the neutralization specificity showed the V2-apex directed neutralization to dominate, with the neutralization specificity of the CAP256-J3 variants clustering closer to CAP256-VRC26.25 than to J3 (Supplementary Fig. 8). This clustering is likely a consequence of the neutralization specificity dependency on the rank order of neutralization, as the CAP256-VRC26.25 parent is more potent though less broad than the J3 parent. On the Seaman clade-C panel, 100% of the 100 clade C strains were neutralized (Figure 5b and Supplementary Table S5). CAP256.J3LS exhibited a geometric mean IC_80_ of 0.05 μg/ml, substantially better than those of CAP256V2LS (0.14 μg/ml), VRC07-523LS (0.42 μg/ml), and N6LS (0.25 μg/ml). Finally, we carried out a manufacturability and biophysical risk assessment, and found CAP256.J3LS to be mostly low risk, except for slight colloidal instability observed at high concentration or with dynamic light scattering (DLS) (Supplementary Figure S9).

## Discussion

In this study, we created a bispecific antibody by linking the light chain of CAP256-VRC26.25 with the J3 nanobody and optimizing half-life to create CAP256.J3LS, a variant with improved breadth, potency, and half-life. CAP256.J3LS compared favorably with other CAP256 variants, such as those obtained by Farzan and colleagues through *in vitro* affinity maturation.^25^ CAP256.J3LS also compared favorably with other engineered bispecific antibodies, as well as with other V2 neutralizers.^26,27^ One of the broadest and most potent V2 neutralizers, PGDM1400,^27^ neutralizes 78% of the 208-strain panel with IC_80_ < 50 μg/ml at a geometric mean IC_80_ of 0.069 μg/ml, whereas CAP256.J3LS neutralized with a breadth of 97% with a geometric mean IC_80_ of 0.035 μg/ml. The bispecific antibody BISC-1C,^26^ which combines CAP256-VRC26.25 with PGT128, neutralizes all 15 strains tested with an IC_80_ of 0.032 μg/ml, whereas CAP256.J3LS neutralized these 15 strains with an IC_80_ of 0.026 μg/ml and neutralized 10 of the 15 strains more potently.

Bispecific antibodies can face manufacturing issues arising from their having two different heavy chain-light chain pairings. A nanobody-antibody bispecific, made by appending the light chain to the C terminus of the nanobody, would bypass these issues because the bispecific antibody would remain symmetric, with both arms having light chain-linked nanobodies. Here, we show that such a bispecific nanobody-antibody can be produced with high yield and, further, can show substantial neutralization synergy, neutralizing better than both antibody and nanobody parental components for over half the 208 viruses tested. Further multimerization, combining CAP256.J3LS with other antibodies, is certainly possible, although, because of the orientation specificities required for synergistic binding, such a multispecific antibody would be unlikely to have other components that bind synergistically with CAP256.J3LS. Nonetheless, such a multispecific antibody would have the advantage of having multiple antibody neutralization specificities in a single product.^28^ It will be interesting to test the impact of CAP256.J3LS clinically.

## Methods

### Antibody expression and purification

Light chain expression constructs of CAP256V2LS and PGDM1400 antibody variants were synthesized (Gene Universal Inc.) and cloned into pVRC8400 expression vector. For the antibody production, 0.15 mL of Turbo293 transfection reagent (Speed BioSystems) were mixed into 2.5 mL Opti-MEM medium (Life Technology) and incubated for 5 min at room temperature (RT). 50 μg of plasmid DNAs (25 μg heavy chain and 25 μg of light chain) were mixed into 2.5 mL of Opti-MEM medium in another tube. The diluted transfection reagent was added into Opti-MEM medium containing plasmid DNAs. Transfection reagents and DNA mixtures were incubated for 15 min at RT and added to 40 mL of Expi293 cells (Life Technology) at 2.5 million cells/ml. The transfected cells were cultured in a shaker incubator at 120 rpm, 37°C, 9% CO_2_ for 5 days. Antibodies in clarified supernatants were purified over 0.5 mL Protein A (GE Health Science) resin in columns. Antibodies were eluted from Protein A columns with a low pH immunoglobulin G (IgG) elution buffer (Pierce) and immediately neutralized with one-tenth volume of 1 M Tris-HCL pH 8.0. The antibodies were buffer exchanged in phosphate-buffered saline (PBS) by dialysis and then the concentration was adjusted to 0.5 mg/ml and filtered (0.22 μm) for neutralization assays. For size-exclusion chromatography analysis, 0.5 mg of 1 mg/ml antibody was injected onto the Superose 6 Increase column (Cytiva Life Sciences) on a BioRad (Hercules, CA) NGC Chromatography System Quest 10. The flow rate was set to 0.5 mL/min, and the mobile phase B was 1 × PBS (Thermo Fisher).

### Expression and purification of HIV-1 Env trimer

A non-tagged HIV Env trimer from the isolate CAP256.wk34.c80 (GenBank: KT698226.1) was stabilized and produced in transiently transfected 293 F cells as previously described.^29,30^ Briefly, 600 μg of the plasmid encoding the trimer and 150 μg the plasmid encoding human furin were mixed and used to transfect 1 L of 293 F cells using Turbo293 transfection reagent (Speed BioSystems). Cells were incubated in shakers at 120 rpm, 37°C, and 9% CO_2_. On the next day, 80 ml HyClone SFM4HEK293 medium and 20 ml FreeStyle™ 293 Expression Medium were added to each liter of cells. The native-like Env trimer protein was purified from the supernatant harvested on day 7 by 2G12 affinity chromatography, followed by gel filtration on a Sephadex200 16/60HL column in PBS.

### Anti-HIV-1 ENV trimer ELISA

Twenty-four hours prior to the DNA-transient transfection, 100 μl/well of log-phase growing HEK 293 T cells were seeded into a flat bottom 96-well tissue culture plate (Corning) at a density of 3 × 10^5^ cells/ml in an optimized expression medium (RealFect-Medium, ABI Scientific) and incubated at 37°C, 5% CO_2_ for 24 hours. Prior to transfection, 40 μl/well of spent medium was removed. For transient transfection, 0.15 ug of heavy-chain plasmid DNA was mixed with 0.15 ug of light-chain plasmid DNA in Opti-MEM medium (Invitrogen), and final volume 10 μl per well in a round bottom 96-well plate, followed by mixing with 10 μl/well of 0.9 μl TrueFect-Max transfection reagent (United BioSystems) in Opti-MEM medium. After incubation for 15 min at RT, 20 μl/well of DNA-TrueFect-Max complex was mixed with growing cells in the 96-well tissue culture plate and incubated at 37°C, 5% CO_2_. At 20 hours post transfection, each well of culture was fed with 30 μl/well of enriched expression medium (CelBooster Cell Growth Enhancer Medium for Adherent Cell, ABI Scientific). After 5 days of transfection, the antibodies in supernatants in the 96-well tissue culture plate were characterized by 96-well-formatted ELISA. Briefly, 96-well ELISA plates (Nunc Maxisorp, Thermo Fisher Scientific) were coated overnight at 4°C with 100 μl/well of 5 μg/ml lectin (Galanthus nivalis, Sigma-Aldrich) in 1× PBS. Between each subsequent step, plates were washed five times with PBS-T (PBS plus 0.05% Tween 20). After being coated, various HIV-1 trimer proteins were captured onto lectin-coated 96-well ELISA plates, respectively, by an incubation of 100 μl/well of 5 μg/ml each trimer protein for 2 hours at RT followed by blocking with 200 μl/well of CelBooster Cell Growth Enhancer Medium for Adherent Cell for 1 hour at RT. After plate wash, 30 μl/well of the expressed antibody supernatant mixed with 70 μl/well of PBS was incubated for 1 hour at RT, then followed by incubation for 30 min at RT with 100 μl/well of horseradish peroxidase (HRP)-conjugated goat anti-human IgG antibody (Jackson ImmunoResearch Laboratories Inc., PA, Cat. No.: 109–035-088), diluted at 1:10,000 in CelBooster Cell Growth Enhancer Medium for Adherent Cell plus 0.02% Tween 20. Finally, the reaction signal was developed with 100 μl/well of tetramethylbenzidine substrate (BioFX-TMB, SurModics) for 10 min at RT before the addition of 100 μl/well of 0.5 N sulfuric acid (Fisher Chemical) to stop the reaction. Plates were read at 450 nm wavelength (SpectraMax using SoftMax Pro, version 5, software; Molecular Devices, Sunnyvale, CA), and the optical densities (OD) were analyzed following subtraction of the nonspecific horseradish peroxidase background activity. All samples were measured in duplicate.

### Virus neutralization assay

As described below, neutralization was assessed in one of four formats of the Env-pseudotyped assay,^31^ all of which yielded highly similar results.

(1) Standard neutralization assays were performed in 96-well formats as follows: 10 μl of five-fold serially diluted mAbs in cDMEM was incubated with 40ul of diluted HIV-1 Env-pseudotyped virus and incubated for 30 minutes at 37°C in a 96-well CulturPlate (Perkin Elmer). 20 μl of TZM-bl cells (10,000 cells/well) with or without 70 μg/ml DEAE-Dextran was then added and incubated overnight at 37°C. Each experiment plate also had a column of cells only (no antibody or virus) and a column of virus only (no antibody) as controls for background TZM-bl luciferase activity and maximal viral entry, respectively. Serial dilutions were performed with a change of tips at each dilution step to prevent carryover.^2^ The following day, all wells received 100 μl of fresh cDMEM and were incubated overnight at 37°C. The following day, 50 μl of Steadylite Plus Reporter Gene Assay System (PerkinElmer) was added to all wells, and plates were shaken at 600RPM for 15 minutes. Luminometry was then performed on a SpectraMax L (Molecular Devices) luminometer. Percent neutralization is determined by calculating the difference in average Relative Light Units (RLU) between virus only wells (cells + virus column) and test wells (cells + plasma/Ab sample + virus), dividing this result by the average RLU of virus only wells (cell + virus column) and multiplying by 100. Background is subtracted from all test wells using the average RLU from the uninfected control wells (cells only column) before calculating the percent neutralization. Neutralizing plasma antibody titers are expressed as the antibody concentration required to achieve 50% neutralization and calculated using a dose–response curve fit with a 5-parameter nonlinear function.

(2) High-throughput screening was performed by single-point assay: supernatants from cells transfected in 96-well plates were used undiluted in the assay described above, in duplicate. Wells with >50% reduction in signal compared to baseline were scored as positive for neutralization.

(3) Selected monoclonal antibodies (mAbs) were assessed on a panel of 208 geographically and genetically diverse Env pseudoviruses representing the major subtypes and circulating recombinant forms.^32^ Assays were performed by microneutralization in an optimized and qualified automated 384-well format as described,^31^ with the modification of changing tips after each antibody dilution. Data were analyzed as above.

(4) Select mAbs were assessed on a panel of 100 clade C Env pseudoviruses from acute infection.^33^ Neutralization assays were conducted using TZM.bl cells as previously described.^31^ Briefly, mAb samples were tested in duplicate in 96-well plates using a primary concentration of 50 µg/ml or 1 µg/ml and serially diluted fivefold seven times. The 1 µg/ml start value was used instead of changing tips, as noted above. The HIV-1 Env pseudovirus was added to antibody serial dilutions and plates were incubated for 1 h at 37°C. TZM.bl cells were then added at 1x10^4^/well with DEAE-Dextran at a final concentration of 11 µg/ml. After 48-h incubation at 37°C, plates were harvested using Bright-Glo luciferase (Promega) and luminescence detected using a GloMax Navigator luminometer (Promega, Madison, WI). Antibody concentrations that inhibited 50% or 80% of viral infection were determined (IC50 and IC80 titers, respectively). Neutralization assays were conducted in a laboratory meeting Good Clinical Laboratory Practice quality assurance criteria.

### Antibody heparin affinity chromatography

Each antibody sample was diluted in 1500 µL of mobile phase A (MPA), 10 mM sodium phosphate, pH 7.2 ± 0.2 to a final concentration of approximately 20 µg/mL. It was then injected onto the HiTrap 1 mL Heparin HP column (Cytiva Life Sciences) on a BioRad (Hercules, CA) NGC Chromatography System Quest 10. The flow rate was set to 1.0 mL/min, and the mobile phase B (MPB) was 10 mM sodium phosphate, 1 M NaCl, pH 7.2 ± 0.2. The column was equilibrated in 100% MPA before each injection; the gradient was as follows (1): 0–2 min, 100% MPA; (2): 2–12 min, 100% MPA to 100% MPB; (3) 12–14 min, 100% MPB. UV absorbance was detected at 280 nm using Chromlab.

### Autoreactivity analysis of antibodies

Autoreactivity of antibodies was evaluated using the ANA Hep-2 Test System (ZEUS Scientific, Cat. No: FA2400) and anticardiolipin ELISA (Inova Diagnostics Cat. No.: 708625). Briefly, all antibodies were tested at 25 and 50 μg/ml as per the protocol from the manufacturer of the ANA Hep-2 Test System. Antibodies VRC01LS, 4E10, VRC07-523-LS, and VRC07-G54W were used as controls and scored as 0, 1, 2, and 3, respectively. HEp-2 cells were obtained from ZEUS Scientific. Slides were imaged on a Nikon Eclipse Ts2R microscope with a 20 × objective lens for 500 ms. The fluorescent signals of the test antibodies were estimated visually in comparison to the control ones. Scores over 1 at 25 μg/ml were defined as autoreactive, and between 0 and 1 as mildly autoreactive. In the cardiolipin ELISA, all the antibodies were tested at 100 μg/ml, followed by a 3-fold serial dilution. IgG phospholipid (GPL) units were derived from the standard curve. GPL score below 20 was considered as not reactive, and between 20 and 80 as low positive and greater than 80 as high positive. The reported results are representative of at least two independent experiments.

### Isothermal titration calorimetry measurement

Binding experiments by ITC were performed at 37°C using a MicroCal VP-ITC microcalorimeter from Malvern Panalytical (Northampton, MA, USA). All reagents were dissolved in and exhaustively dialyzed against PBS, pH 7.4. The syringe was filled with either J3 nanobody at a concentration of ~0.3 mg/mL (~24 µM) or either CAP256V2LS IgG or bispecific CAP256.J3LS IgG at ~1.0 mg/mL (~6 µM IgG). The calorimetric cell was filled with BG505 DS-SOSIP.664 Env trimer at a concentration of ~0.2 mg/mL (~1 µM trimer) and either an IgG or a nanobody was added stepwise in 6 or 10 µL aliquots with 300 s interval during a continuous stirring at 300 rpm. The heat evolved upon each injection was obtained from the integral of the calorimetric signal, and the heat associated with binding was obtained after subtraction of the heat of dilution. The enthalpy change, ΔH, the association constant, Ka (the dissociation constant, Kd = 1/Ka) and the stoichiometry, N, were obtained by nonlinear regression of the data to a single-site binding model using Origin with a fitting function made inhouse. The Gibbs energy, ΔG, was calculated from the binding affinity using ΔG = -RTlnKa (R = 1.987 cal/(K × mol)) and T is the absolute temperature in kelvin. The entropy contribution to the Gibbs energy, -TΔS, was calculated from the relation ΔG = ΔH -TΔS. The results were normalized per mole of nanobody or half-IgG and the stoichiometry, N, denotes the number of half-IgG per trimer.

### Pharmacokinetic study in human neonatal Fc receptor (FcRn) transgenic mice

Human FcRn transgenic mice (FcRn-/- hFcRn (32) Tg mice, JAX stock #014565, The Jackson Laboratory) were used to assess the pharmacokinetics of wild type and Fv glycan-removed antibodies. Each animal was infused intravenously with 5 mg mAb/kg of body weight. Whole blood samples were collected at day 1, 2, 5, 7, 9, 14, and 21. Serum was separated by centrifugation. Serum mAb levels were measured by ELISA using either anti-idiotypic antibodies (for VRC01 LS; CAP256-VRC26.25, CAP256V2LS, CAP256V2LS-J3-3, or CA256.J3LS) as described previously.^34^ All mice were bred and maintained under pathogen-free conditions at the American Association for the Accreditation of Laboratory Animal Care-accredited Animal Facility at the National Institute of Allergy and Infectious Diseases and housed in accordance with the procedures outlined in the Guide for the Care and Use of Laboratory Animals. All the mice were between 6 and 13 weeks of age. The study protocol was evaluated and approved by the National Institutes of Health’s Animal Care and Use Committee (ASP VRC-18-747).

### Neutralization fingerprinting analysis

The neutralization fingerprint of an mAb or polyclonal plasma is defined as the potency pattern with which the antibody/plasma neutralizes a set of diverse viral strains. The neutralization fingerprints of 46 mAbs, including all described CAP256.VRC26.25 variants, were compared and clustered according to fingerprint similarity, as described previously.^35^ A set of 208 HIV-1 strains was used in the neutralization fingerprinting analysis.

### Cryo-EM data collection and processing

The BG505 DS-SOSIP.664 Env trimer^20,21^ was incubated with a molar excess of CAP256V2LS-J3-3 bispecific antibody Fab fragments. A volume of 2.3 µl of the complex at 2 mg/ml concentration was deposited on a C-flat 1.2/1.3 grid (protochip.com) and vitrified with an FEI Vitrobot Mark IV with a wait time of 30 seconds, blot time of 3 seconds, and blot force of 1. Data collection was performed on a Titan Krios electron microscope with Leginon^36^ using a Gatan K3 direct detection device. Exposures were collected in movie mode for 2 seconds with the total dose of 51.19 e^–^/Å^2^ fractionated over 50 raw frames. cryoSPARC^37^ v3.3 was used for frame alignment, CTF estimation, 2D classifications, ab initio 3D reconstruction, homogeneous refinement, and nonuniform 3D refinement. Initial 3D reconstruction and final refinements were performed using C1 symmetry. The final resolution of the C1 non-uniform refinement was 3.18 Å.

Coordinates from the PDB IDs 6VTT^7^ and 7LPN^15^ were used for initial fits to the reconstructed map. This was followed by simulated annealing and real space refinement in Phenix v1.20^38^ with the sharpened map from cryoSPARC v3.3 and with a density modified map from Phenix Resolve^39^ and manually fit with Coot^40^ v0.9.8 and then improved through iterative rounds. Geometry and map fitting parameters were evaluated using Molprobity^41^ v4.5.1 and EMRinger.^42^ PyMOL v2.5 (www.pymol.org) and ChimeraX^43^ v1.3 were used to generate figures.

### Manufacturability assessment

Manufacturability for CAP256.J3LS was assessed by visual inspection, dynamic light scattering, thermal transitions by dynamic light scattering, differential scanning calorimetry, circular dichroism, and isothermal chemical denaturation as previously described.

## Supplementary Material

Supplemental Material

## Data Availability

Cryo-EM maps and fitted coordinates have been deposited with EMDB entry ID EMD-29209 (https://www.ebi.ac.uk/emdb/search/EMD-29209) and PDB entry ID 8FIS (https://www.rcsb.org/structure/unreleased/8FIS), respectively.

## Abbreviations

AMP, antibody-mediated prevention; CDR, complementarity determining region; DLS, dynamic light scattering; Env, HIV-1 envelope glycoprotein; Fab, antigen-binding fragment; FcRn, neonatal Fc receptor; IC_50_, half maximal inhibitory concentration; IC_80_, 80% inhibitory concentration; IgG, immunoglobulin G; ITC, isothermal titration calorimetry; MPER, membrane-proximal external region; OD, optical density; RT, room temperature; scFv, single-chain variable region; V2, second hypervariable region
