# Supplementary figures and images for: Bispecific antibody CAP256.J3LS targets V2-apex and CD4-binding sites with high breadth and potency
Source: MAbs. 2023 Feb 2;15(1):2165390. doi: 10.1080/19420862.2023.2165390 (PMC9897750; doi:10.1080/19420862.2023.2165390)

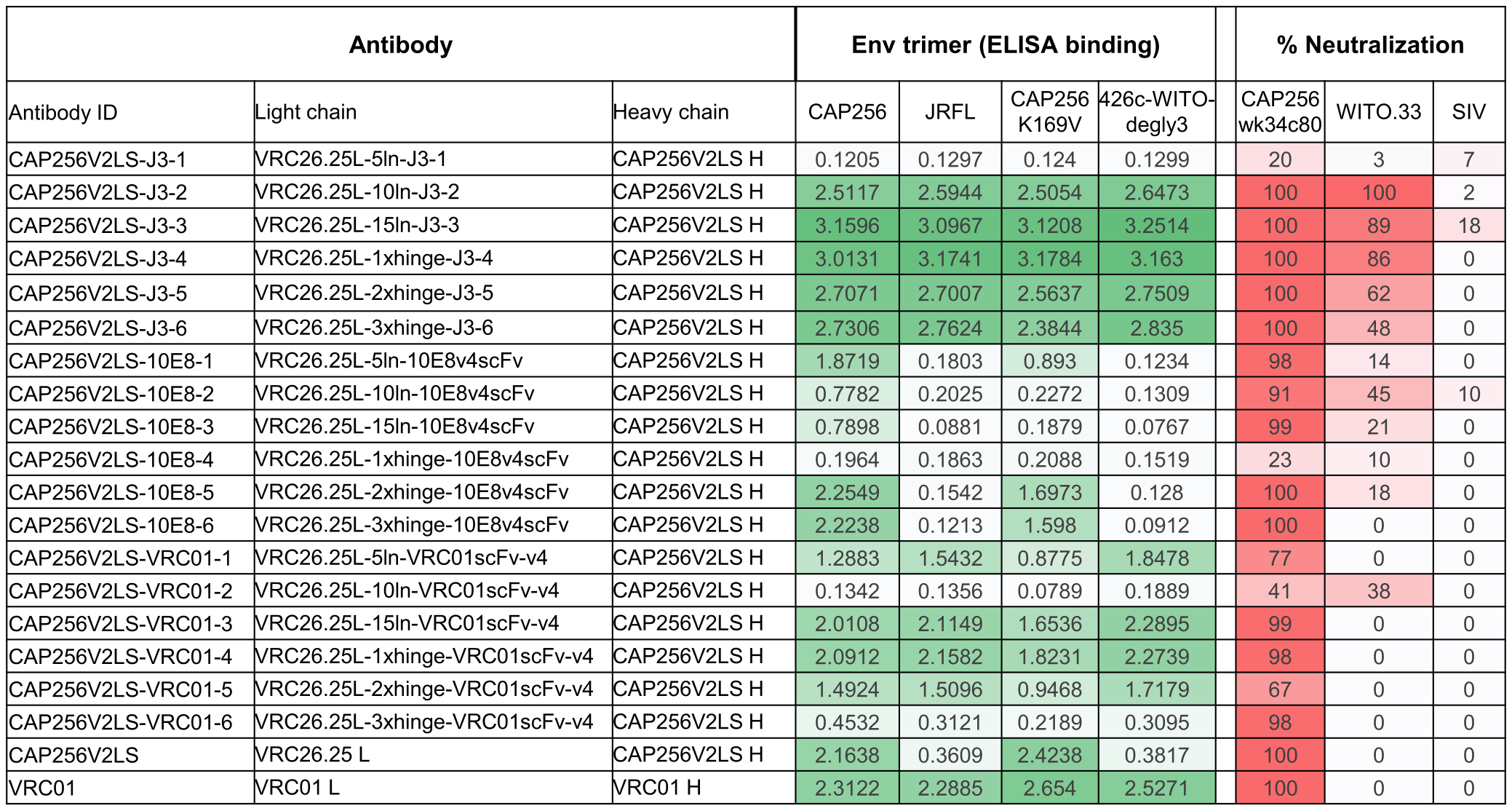

Supplement: Supplemental Material [file KMAB_A_2165390_SM9733.zip › SuppFig1_KMAB20220170_20221222.tif]

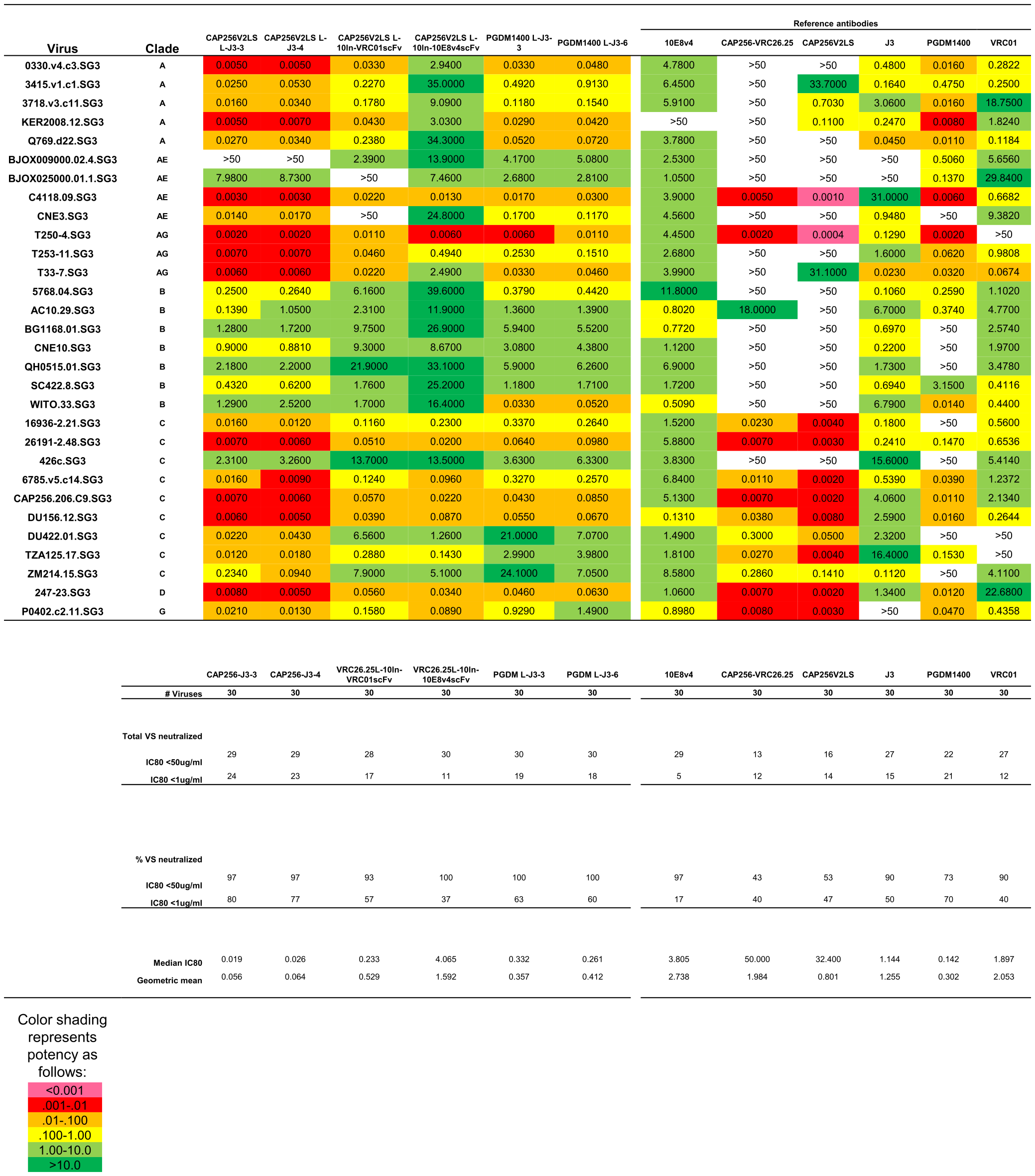

Supplement: Supplemental Material [file KMAB_A_2165390_SM9733.zip › SuppFig2_KMAB20220170_20221222.tif]

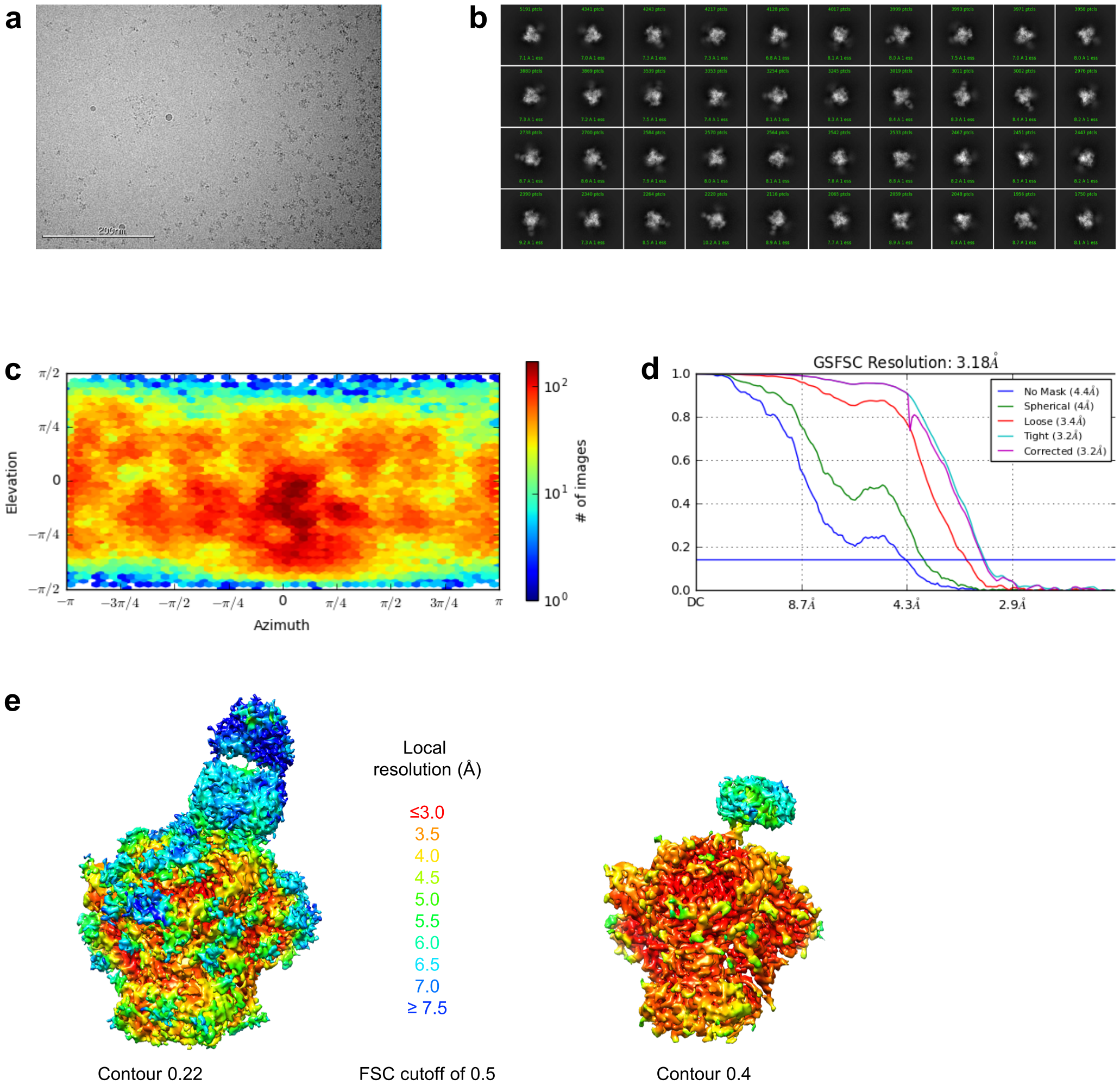

Supplement: Supplemental Material [file KMAB_A_2165390_SM9733.zip › SuppFig3_KMAB20220170_20221222.tif]

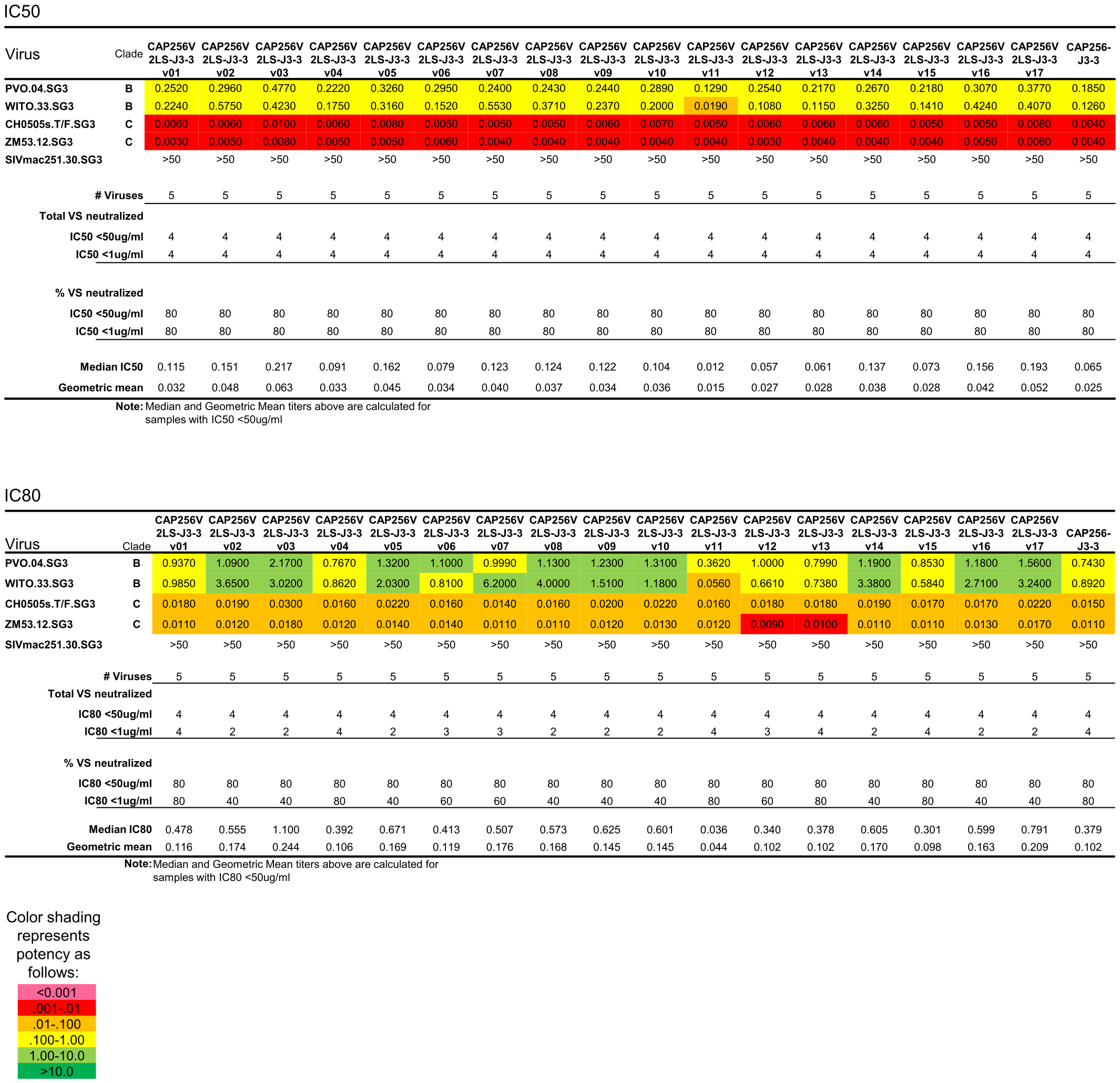

Supplement: Supplemental Material [file KMAB_A_2165390_SM9733.zip › SuppFig4_KMAB20220170_20221222.tif]

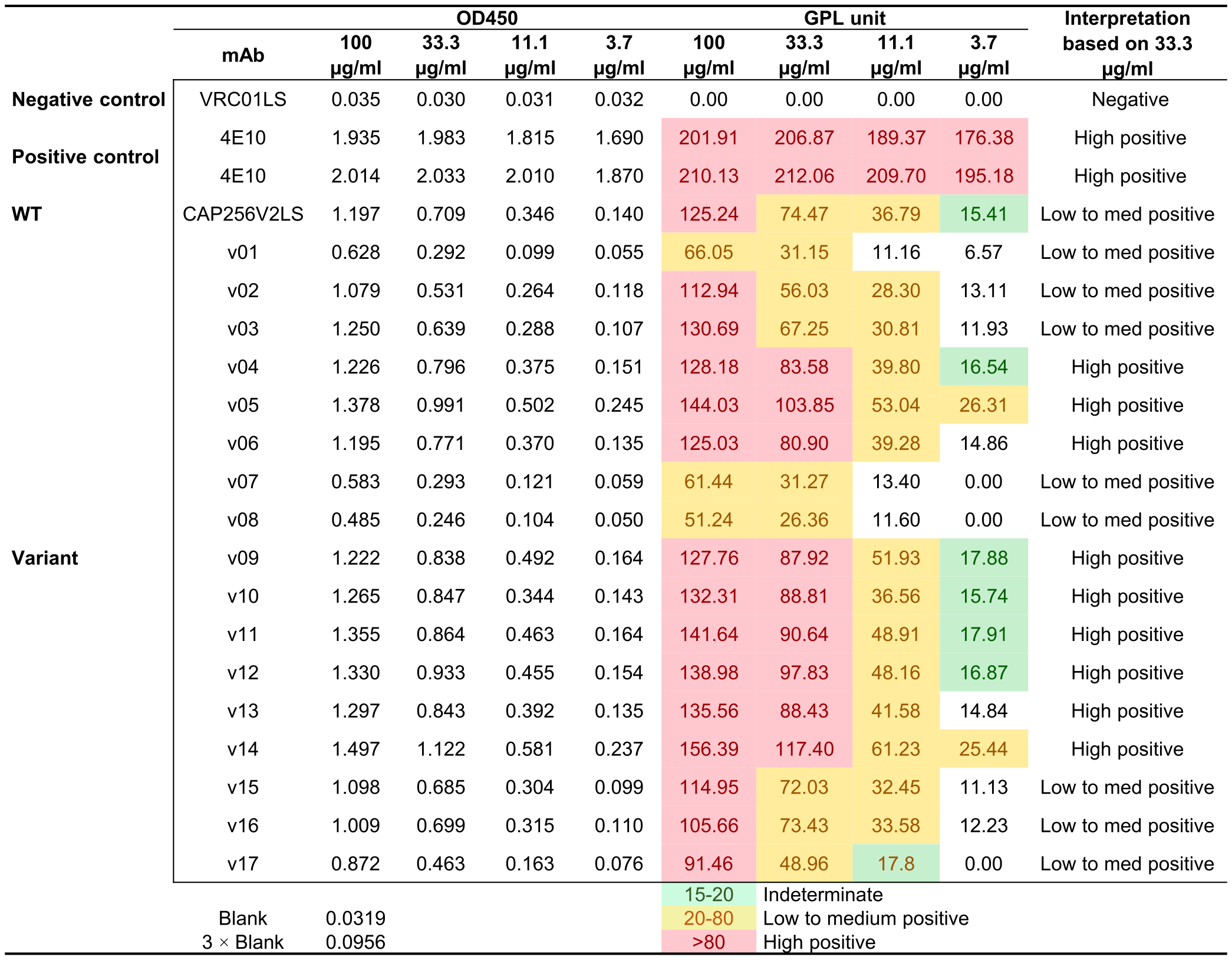

Supplement: Supplemental Material [file KMAB_A_2165390_SM9733.zip › SuppFig5_KMAB20220170_20221222.tif]

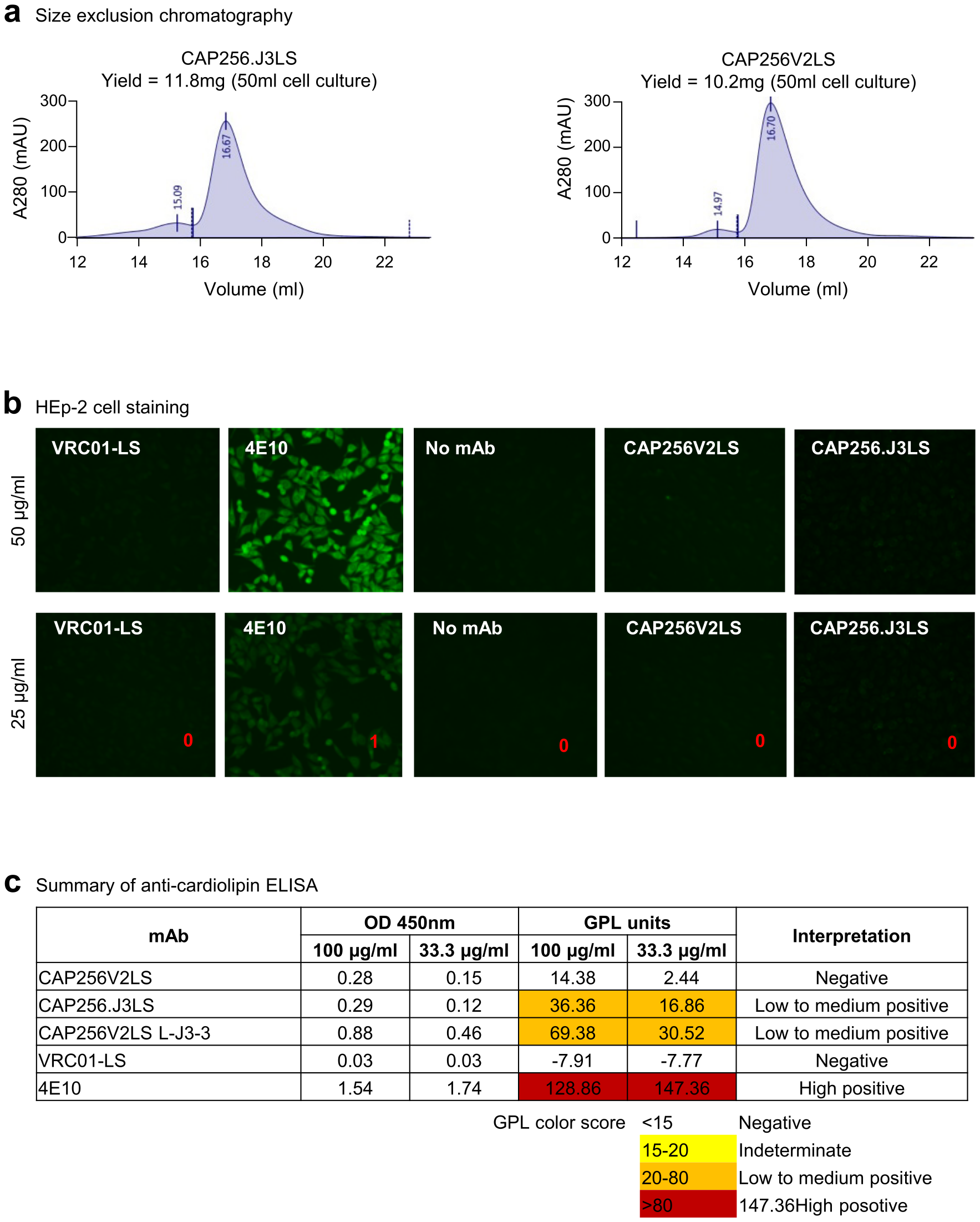

Supplement: Supplemental Material [file KMAB_A_2165390_SM9733.zip › SuppFig6_KMAB20220170_20221222.tif]

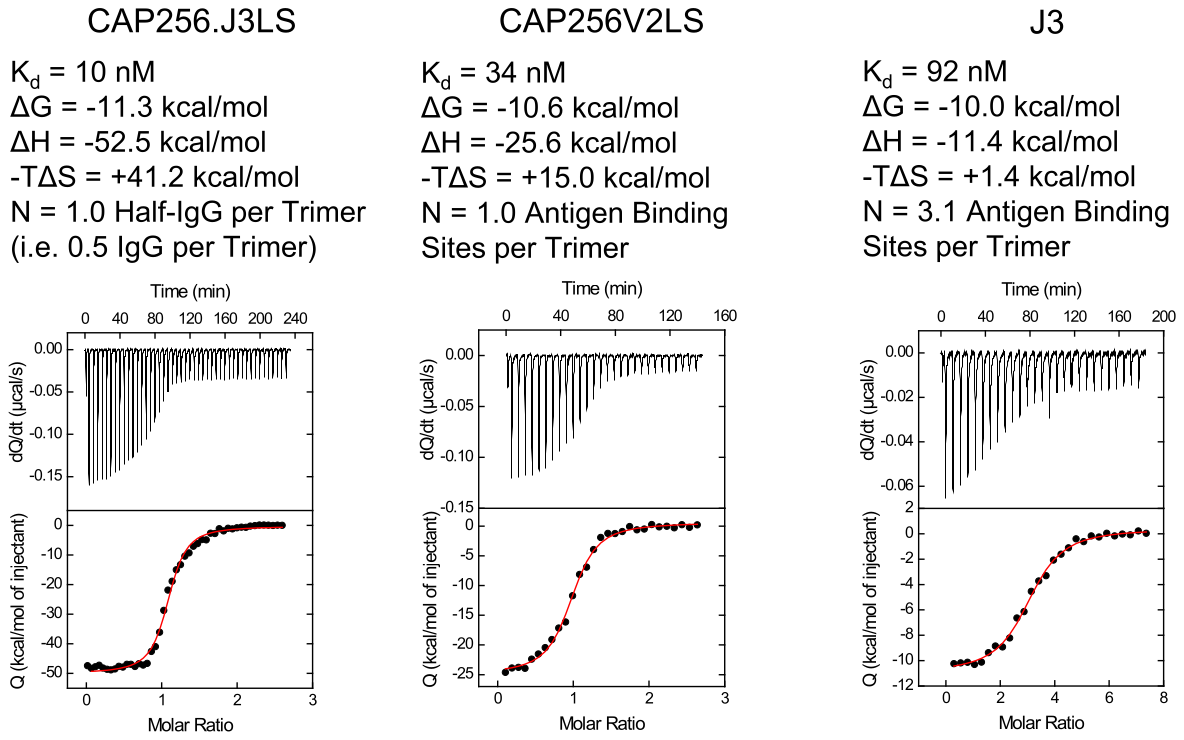

Supplement: Supplemental Material [file KMAB_A_2165390_SM9733.zip › SuppFig7_KMAB20220170_20221222.tif]

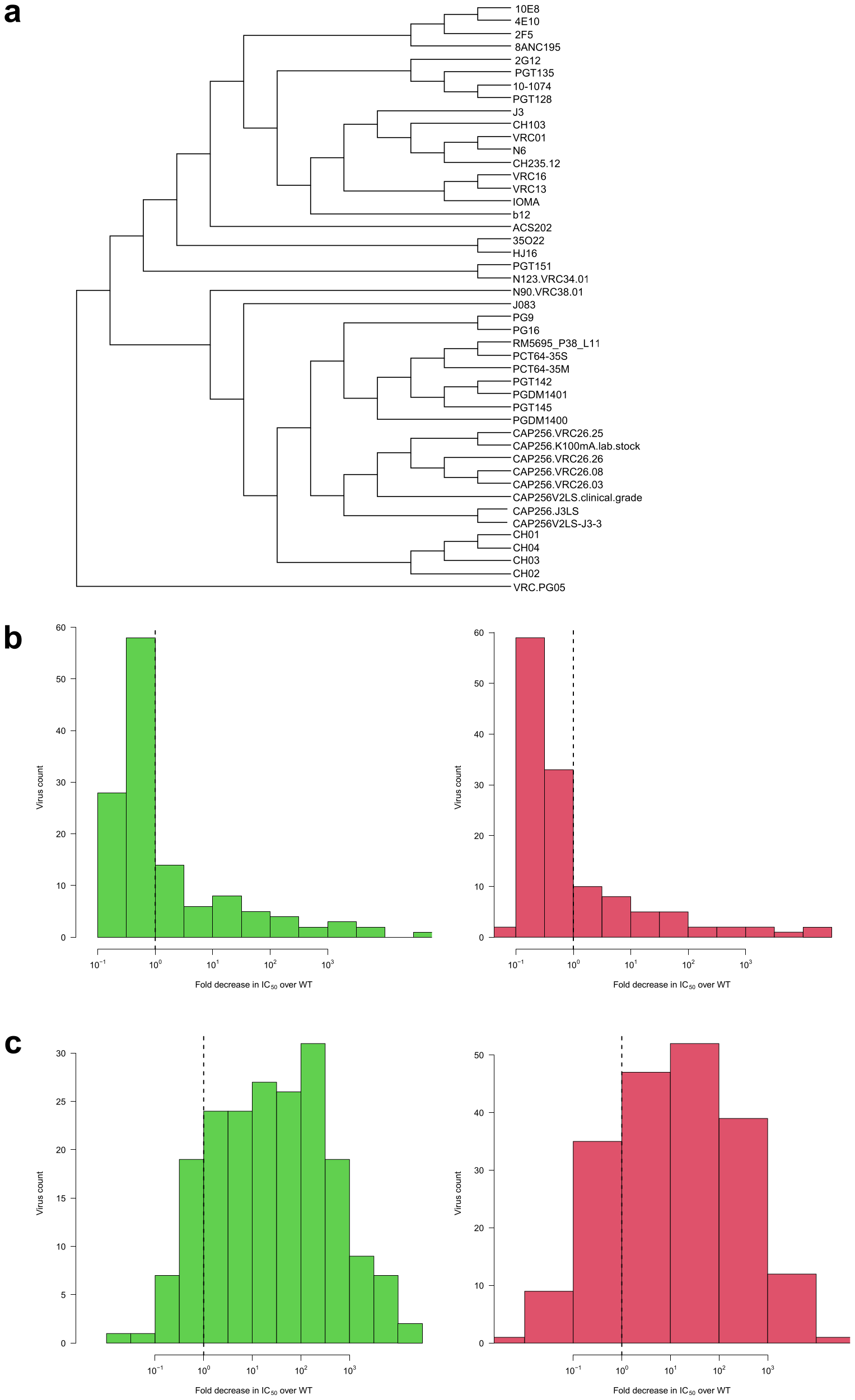

Supplement: Supplemental Material [file KMAB_A_2165390_SM9733.zip › SuppFig8_KMAB20220170_20221222.tif]

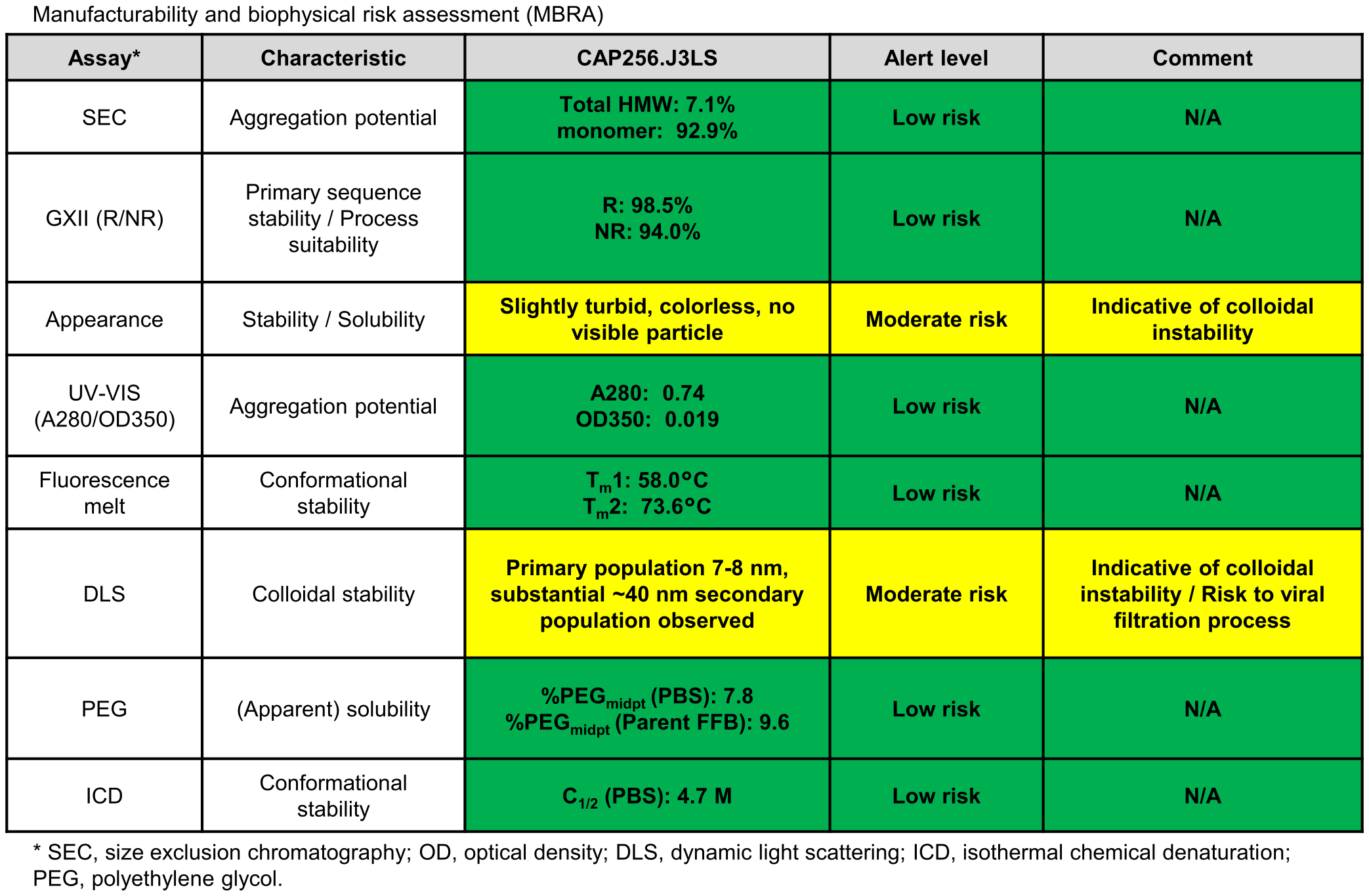

Supplement: Supplemental Material [file KMAB_A_2165390_SM9733.zip › SuppFig9_KMAB20220170_20221222.tif]
